# Avaliação dos Efeitos Cardíacos de Lectina Solúvel em Água (WSMoL) de Sementes de Moringa Oleifera

**DOI:** 10.36660/abc.20190071

**Published:** 2020-06-29

**Authors:** Ainhoa Rodríguez de Yurre, José Dayvid Ferreira da Silva, Marília Kalinne da Silva Torres, Eduarda Lopes Martins, Isalira Peroba Ramos, Wênio Sandoval Filho Lima da Silva, Jéssica da Silva Sarpa, Caio César da Silva Guedes, Thiago Henrique Napoleão, Luana Cassandra Breitenbach Barroso Coelho, Patrícia Maria Guedes Paiva, Emiliano Medei

**Affiliations:** 1 Universidade Federal do Rio de Janeiro Rio de JaneiroRJ Brasil Universidade Federal do Rio de Janeiro, Rio de Janeiro, RJ – Brasil; 2 Universidade Federal de Pernambuco RecifePE Brasil Universidade Federal de Pernambuco, Recife, PE – Brasil

**Keywords:** Moringa Oleifera, Lectinas, Glicosídeos, Carboidratos, Coração, Segurança Hídrica, Camundongos

## Abstract

**Fundsamento:**

As sementes de *Moringa oleifera* , que são utilizadas para clarificação de água, contêm uma lectina chamada WSMoL que tem mostrado atividade antibacteriana e imunomoduladora *in vitro* . Devido ao seu valor nutritivo e potencial terapêutico, as folhas e as sementes dessa árvore são consumidas em algumas comunidades. Algumas lectinas de plantas não são tóxicas para mamíferos, mas tem sido relatado que outras são prejudiciais quando ingeridas ou administradas por outros meios.

**Objetivo:**

Como um dos passos necessários para determinar a segurança de WSMoL, nós avaliamos os possíveis efeitos cardiotóxicos desta proteína purificada.

**Métodos:**

Durante 21 dias consecutivos, a WSMoL foi administrada a camundongos por gavagem. Foram investigadas as funções eletrofisiológicas, mecânicas e metabólicas *in vivo* e *ex vivo* por meio de registros eletrocardiográficos, ressonância magnética nuclear e respirometria de alta resolução.

**Resultados:**

O tratamento com WSMoL não induziu alterações nos níveis de glicose no sangue ou peso corporal em comparação com o grupo controle. Adicionalmente, as relações peso cardíaco/peso corporal e peso cardíaco/comprimento tibial estavam semelhantes em ambos os grupos. A ingestão de lectina também não modificou a tolerância à glicose ou resistência à insulina. Não foram observadas alterações nos parâmetros eletrocardiográficos ou na duração do potencial de ação cardíaco. Os corações dos camundongos dos grupos controle e WSMoL mostraram função ventricular esquerda preservada. Além disso, a WSMoL não induziu alterações na função mitocondrial (em todos os casos, p > 0,05).

**Conclusões:**

A administração de WSMoL demonstrou ter um perfil de segurança cardíaca. Estes resultados contribuem à avaliação de segurança do uso de sementes de *M. oleifera* para tratar água, visto que essa lectina está presente na preparação empregada por algumas populações com esse fim. (Arq Bras Cardiol. 2020; [online].ahead print, PP.0-0)

## Introdução

*Moringa oleifera* Lamarck (Moringaceae) é uma árvore nativa da região sul do Himalaia, amplamente cultivada na Ásia e nos trópicos, principalmente devido ao seu uso para clarificação de água. Tem sido cultivada como um remédio tradicional, utilizada nas indústrias alimentar, cosmética e farmacêutica,^1 , 2^ e também é usada para tratar várias doenças, como câncer e doenças crônicas e infecciosas.^3 , 4^

Uma lectina solúvel em água isolada das sementes de *M. oleifera* (WSMoL) tem demonstrado atividade inseticida,^5 - 7^ e estudos *in vitro* demonstraram sua atividade antibacteriana contra bactérias corrosivas e patogênicas.^8 - 10^ A WSMoL demonstrou atividade anti-inflamatória *in vitro* em macrófagos murinos estimulados por lipopolissacarídeos,^11^ e foi capaz de ativar linfócitos humanos a partir de culturas de células mononucleares do sangue periférico, mostrando um efeito imunomodulador.^12^ Também tem sido comprovado que WSMoL é uma das proteínas coagulantes encontradas nas sementes de *M. oleifera* ,^8 , 13^ e é capaz de reduzir a turbidez e a ecotoxicidade de amostras de água coletadas de um riacho poluído.^14^

Está bem demonstrado que muitos antibióticos e algumas classes de drogas anti-inflamatórias estão frequentemente associados a efeitos cardiotóxicos.^15 , 16^ Entre os eventos adversos no sistema cardiovascular, estão a ocorrência de insuficiência cardíaca com disfunção ventricular sistólica, arritmias e isquemia miocárdica.^17^ Classicamente, como uma consequência de cardiotoxicidade, podem ser observadas alterações no eletrocardiograma (ECG), tais como o prolongamento do intervalo QT, o qual tem sido observado em pacientes que usaram várias classes de drogas antimicrobianas, incluindo macrólidos e fluoroquinolonas.^18 - 20^ Entre os macrólidos, a administração intravenosa de eritromicina apresenta o risco maior de aumento do intervalo QT, e arritmias fatais têm sido relatadas quando foi usada isoladamente ou em combinação com outras drogas que prolongam o intervalo QT.^16^ Portanto, a proteção da função cardíaca atualmente está um desafio constante para a indústria farmacêutica, autoridades reguladoras e médicos que enfrentam reações clínicas adversas de vários agentes terapêuticos na prática clínica.

WSMoL tem surgido como um potencial medicamento antibacteriano e como um agente imunomodulador. Algumas lectinas de plantas não são tóxicas para mamíferos,^21 , 22^ enquanto outras têm sido relatadas como prejudiciais quando ingeridas ou administradas por outros meios, como injeção intraperitoneal.^23^ Portanto, como um dos passos necessários para determinar a segurança de WSMoL, este estudo avaliou os possíveis efeitos cardiotóxicos desta proteína.

## Métodos

### Material das plantas e isolação da lectina

Foram coletadas sementes de *moringa oleifera* em Recife (Pernambuco, Brasil) com a autorização (nº 38690) do Instituto Chico Mendes de Conservação da Biodiversidade (ICMBio) e armazenadas a −20 ºC. Uma amostra do material coletado foi armazenada como um atestado de espécie (número 73345) no herbário Dárdano de Andrade Lima do Instituto Agronômico de Pernambuco. Foi registrado o acesso (A6CAB4C) no Sistema Nacional de Gestão do Patrimônio Genético e do Conhecimento Tradicional Associado (SisGen).

A WSMoL foi isolada a partir do pó das sementes de acordo com o protocolo previamente descrito por Coelho et al.,^5^ Resumidamente, as proteínas foram extraídas em água destilada, e, após filtração e centrifugação, o extrato foi tratado com sulfato de amônio com saturação de 60%^24^ durante 4 h a 28 °C. Após outra centrifugação, o precipitado foi ressuspenso em água e dialisado durante 8 h contra água destilada (4 h) e NaCl 0,15 M (4 h). A fração dialisada (100 mg de proteínas) foi carregada em uma coluna de quitina equilibrada com NaCl 0,15 M (taxa de fluxo de 20 mL/h), e o WSMoL foi eluído com 1,0 M de ácido acético. A lectina isolada foi dialisada contra água destilada com três trocas de líquido para eliminação do eluente. A atividade de ligação de carboidratos da lectina foi monitorada durante o processo de purificação pelo ensaio da atividade hemaglutinante, de acordo com o método descrito por Paiva e Coelho.^25^

### Animais

Foram utilizados camundongos machos adultos C57BL/6 mantidos no Instituto de Biofísica Carlos Chagas Filho (IBCCF) da Universidade Federal do Rio de Janeiro (UFRJ) sob condições controladas de temperatura constante (23 °C), com um ciclo claro/escuro de 12h/12h e acesso livre a comida e água. Todos os experimentos foram realizados de acordo com os Princípios Éticos de Pesquisa em Animais adotados pelo Colégio Brasileiro de Experimentação Animal, e os protocolos aplicados foram aprovados pelo Comitê de Ética em Pesquisa em Animais da UFRJ, sob número de protocolo DFBCICB041. Os camundongos foram utilizados para experimentos durante 21 dias.

### Condições experimentais

Os animais foram separados em dois grupos experimentais: CNTRL (grupo controle) e WSMoL (animais tratados com WSMoL). Vários estudos do nosso grupo têm extensivamente realizado experimentos com WSMoL utilizando concentrações entre 10 μg/ml e 0,2 mg/ml^5 - 12^ com a finalidade de testar diversos efeitos biológicos de WSMoL. No presente estudo, para testar a cardiotoxicidade desta proteína purificada, foi utilizada uma concentração de WSMoL 10 vezes mais alta. Deste modo, os animais do grupo WSMoL foram tratados com a lectina (proteína purificada) por gavagem, com uma concentração 5 mg/kg do peso corporal (equivalente a 2 mg/ml) durante 21 dias. Os animais no grupo CNTRL foram tratados com água milli-Q por gavagem durante 21 dias.

### Hipertrofia cardíaca

Com a finalidade de avaliar a existência de possível hipertrofia cardíaca, os corações dos camundongos foram pesados, e os dados foram normalizados, calculando as relações peso cardíaco/peso corporal (PC/PCorp) e peso cardíaco/comprimento tibial (PC/CT).^26 , 27^ Após a pesagem, os animais foram sacrificados por meio de deslocamento cervical. Subsequentemente, os corações foram extraídos, lavados com solução salina tamponada com fosfato (PBS), secados para remover o excesso de líquido e pesados. O comprimento tibial foi medido com um paquímetro.

### Glicemia de jejum, teste de tolerância à glicose intraperitoneal e teste de tolerância à insulina intraperitoneal

As concentrações de glicemia de jejum foram determinadas a partir do sangue das veias da cauda usando um glicosímetro automatizado (Contour^TM^ TS Bayer), teste de tolerância à glicose intraperitoneal (TTGI) e o teste de tolerância à insulina intraperitoneal (TTII), os camundongos foram mantidos em jejum por 6 h e 4 h, respectivamente. Após o período de jejum, os animais receberam, por via intraperitoneal, 2 g/kg de glicose para o TTGI ou 0,5 IU/kg de insulina TTII,^28^ e níveis de glicemia de jejum foram monitorizados 0, 15, 30, 60, 120 min após injeção de um corte na cauda. Foi calculada a área sob a curva (AUC) utilizando todos os pontos no tempo, descontando os valores basais de glicose para cada animal.

### Eletrocardiografia e ecocardiografia

Para avaliar a atividade elétrica cardíaca *in vivo* , foi realizado um registro de eletrocardiograma (ECG) nos animais conscientes utilizando um método não invasivo,^29^ a saber: dois eletrodos subcutâneos implantados sob anestesia com isoflurano nas patas dianteiras direita e esquerda, correspondendo à derivação I do ECG. No momento do registro, os eletrodos foram conectados por cabos flexíveis a um amplificador diferencial caseiro acoplado em CC (generosamente fornecido por Dr. Ariel Escobar, University of California, Merced, EUA), utilizando um filtro passa-baixo de 500 Hz e uma frequência de aquisição de 1 kHz. O sinal foi digitalizado usando Digidata 1440A (Axon Instruments, San José, CA, EUA) e registrado usando um programa de aquisição baseado em Labview (National Instruments, Austin, TX, EUA). Foram analisadas as durações dos seguintes intervalos: PR, RR, QRS e QJ.

A função cardíaca foi avaliada por ecocardiografia (ECHO) *in vivo* utilizando o Sistema de Imagem de Alta Resolução Vevo 770 (VisualSonics, Toronto, Canadá) acoplado a um transdutor de 30 MHz, sob anestesia com isoflurano. As imagens foram adquiridas na modalidade bidimensional e analisadas por um investigador cego. Foram calculados o volume diastólico final, o volume sistólico final, a fração de ejeção e a mudança de área fracionada do ventrículo esquerdo utilizando o método de Simpson. Resumidamente, estes parâmetros de função cardíaca foram avaliados em um corte do eixo paraesternal longitudinal e em quatro imagens do eixo curto no modo B em alta resolução temporal, obtidas em diferentes níveis ventriculares, como descrito anteriormente.^30^

### Potencial de ação

Para realizar registros do potencial de ação (PA) cardíaco em coração intato, um sistema de perfusão retrógrada de Langendorff foi utilizado para manter os corações funcionais ex vivo durantes horas, como previamente descrito.^31 , 32^ Para evitar danos no tecido cardiaco pela formação de coágulos sanguíneos, os animais foram injetados por via intraperitoneal, com Na^+^-heparina, 15 min antes da eutanásia que foi realizada por deslocamento cervical. Os corações foram rapidamente removidos, canulados pela aorta e continuamente perfundidos com uma solução de Tyrode oxigenada contendo o seguinte (em mM): NaCl 140, KCl 5,3, CaCl_2_ 2, MgCl_2_ 1, NaPO4H2 0,33, HEPES 10 e glicose 10. O pH foi calibrado para 7,4 com NaOH a 32 ºC. Para diminuir a contração mecânica, os corações foram perfundidos com Tyrode contendo 4 mM de Blebbistatin (Selleckchem, Houston, TX, EUA).

Foram usados microeletrodos de vidro borossilicato (10-40 MΩ) para registrar os sinais elétricos. Estes microeletrodos foram preenchidos com solução de KCl 3 M e inseridos em um suporte (MEH1SF12, World Precision Instrument [WPI], Sarasota, FL, EUA) incorporado em um micromanipulador (MM33 links, WPI) conectado à entrada de um pré-amplificador (Electro 705, WPI). Os microeletrodos foram colocados na superfície do ventrículo esquerdo e a leitura do microeletrodo foi ajustada em zero. Os sinais amplificados foram digitalizados (NI USB 6281, National Instrument) e analisados com um programa caseiro em LabView (generosamente desenvolvido e fornecido por Dr. Ariel Escobar, University of California, Merced, CA, EUA).

Os parâmetros analisados foram a duração do potencial de ação (DPA) a 30% e 90% de repolarização (DPA_30_ e DPA_90_, respectivamente).

### Isolamento das mitocôndrias cardíacas dos camundongos

O isolamento das mitocôndrias cardíacas dos camundongos foi adaptada do protocolo descrito por Affourtit et al.,^33^ com pequenas modificações. Os corações foram rapidamente dissecados e lavados em tampão Chappell-Perry (CP) gelado, contendo o seguinte (em mM): KCl 100, Tris-HCl 50, EGTA 2 com pH de 7,2. Os corações foram pesados, picados com lâminas e lavados 4 a 5 vezes com tampão CP. O tecido foi subsequentemente incubado por 5 min com tampão CP suplementado com albumina a 0,5%, 5 mM de MgCl2, 1 mM de ATP e 125 U/100 mL de protease tipo VIII, na proporção de 1 mL/100 mg de tecido. Após isso, os corações foram homogeneizados (Ultra-turrax homogenizer [IKA®, Campinas, SP, Brasil], configuração baixa, 3 s, 3 vezes), e o homogenato resultante foi centrifugado. O sobrenadante foi centrifugado e o sedimento foi lavado e ressuspenso em tampão CP gelado e finalmente centrifugado. O sedimento mitocondrial final foi ressuspenso em um volume pequeno de tampão CP. A dosagem de proteína da preparação obtida foi realizada pelo método descrito por Lowry et al.^34^ . As preparações mitocondriais isoladas foram submetidas à respirometria de alta resolução para medir os fluxos de consumo de oxigênio.

### Respirometria de alta resolução

Para as análises de consumo de oxigênio, foram usadas mitocôndrias isoladas. Os experimentos foram realizados em um respirômetro O2k de alta resolução (Oroboros Instruments, Innsbruck, Austria, UE) a 37°C com meio de respiração mitocondrial (MIR05) contendo o seguinte (in mM): EGTA 0.5, MgCl_2_ 3, K-MES 60, taurina 20, KH_2_PO_4_ 20, HEPES 20, sacarose 110 e 1 g/L BSA livre de gordura com pH de 7,1. O protocolo utilizado para avaliar a função mitocondrial foi adaptado de Pesta e Gnaiger,^35^ consistindo na adição sequencial de múltiplos substratos e inibidores, a saber: 5 mM piruvato, 2,5 mM malato, 10 mM glutamato, 100 μM 5’-difosfato de adenosina (ADP), 1 mM ADP, 10 mM succinato, 0,2 μg/mL oligomicina e 2 μM antimicina A. A relação de controle respiratório (RCR) foi calculada pelo fluxo de oxigênio após a adição do succinato na presença de ADP, dividido pelo fluxo após a oligomicina. A capacidade fosforilativa máxima do sistema de transporte de elétrons (OXPHOS) foi calculado pelo consumo de oxigênio após a adição do succinato menos o consumo de oxigênio residual (ROX), o qual foi estimado após a adição da antimicina A. O vazamento inespecífico de prótons foi determinado pelo fluxo de oxigênio não sensível à oligomicina menos o ROX. Um protocolo distinto foi realizado, alterando a sequência dos substratos, para calcular o vazamento de elétrons, a relação de peróxido de hidrogênio (H_2_O_2_) pelo fluxo de O_2_. A ordem de titulação deste protocolo foi a seguinte: 5 mM piruvato, 2.5 mM malato, 10 mM glutamato, 10 mM succinato, 1 mM ADP e 0,2 μg/mL oligomicina. Os dados foram analisados em software DatLab 5 (Oroboros Instruments) e expressos em pmol O_2_/mg/s.

### Produção mitocondrial de H2O2

O H_2_O_2_ mitocondrial foi medido monitorizando-se a taxa de aparecimento de resorufina a 563/587 nm (excitação/emissão) em um espectrofotômetro de fluorescência (Varian Cary Eclipse, Agilent Technologies, Santa Clara, CA, EUA). A mesma concentração de mitocôndria isolada que foi utilizada nos experimentos de consumo de oxigênio foi acrescentada em 2 mL de MIR05 suplementado com 5,5 μM Amplex red, 2 U/mL peroxidase e 40 U/mL superóxido dismutase. Os ensaios de produção de H_2_O_2_ foram realizados a 37 ºC, e os substratos, inibidores e desacopladores foram acrescentados na ordem seguinte: 5 mM piruvato, 2,5 mM malato, 10 mM glutamato, 10 mM succinato, 1 mM ADP, 0,2 μg/mL oligomicina, 2 títulos de 0,5 μM de cianeto de carbonila-4-(trifluorometoxi) fenil-hidrazona (FCCP) e 2 μM antimicina A. Os dados gerados em unidades arbitrárias de fluorescência foram analisados no software Origin Pro-8 (Origin Lab Corporation, Northampton, MA, EUA) e normalizados em pmol de H_2_O_2_/mg/min a partir da calibração padrão das curvas de H_2_O_2_ realizadas na presença do mesmo número de mitocôndrias isoladas para cada experimento.

### Análise estatística

Os valores são expressos como média ± desvio padrão ou mediana (com intervalo interquartil). Para comparar os resultados entre os grupos CNTRL e WSMoL, foi utilizado o teste t de Student não pareado, quando apropriado. De outra maneira, os dados que apresentaram distribuição não gaussiana (teste Kolmogorov-Smirnov) foram comparados pelo teste Mann-Whitney. Foram consideradas significativas as diferenças entre as variáveis quando o valor p era < 0,05. Todas as análises foram realizadas utilizando GraphPad Prism 7.0 (GraphPad Software, San Diego, CA, EUA). Nós não utilizamos métodos estatísticos para predeterminar o tamanho das amostras. Os tamanhos das amostras foram estimados com base na disponibilidade da amostra e em estudos experimentais anteriores do sistema cardiovascular.^29 , 30^

## Resultados

**Figura 1:**
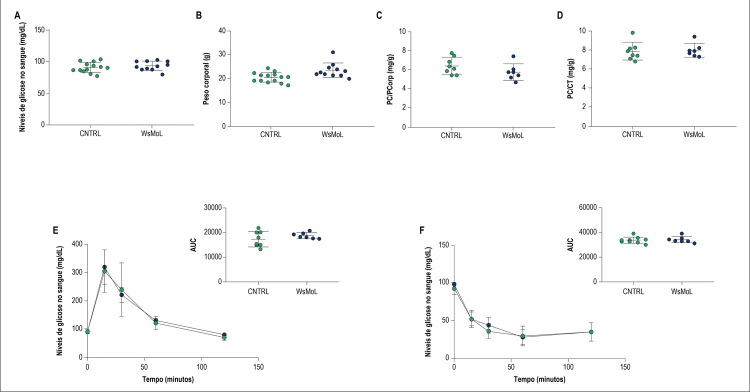
– Tratamento com WSMoL durante 21 dias não induziu alterações metabólicas. (A) Níveis de glicose no sangue após 21 dias de tratamento com solução salina (CNTRL) ou solução de WSMoL (WSMoL) (CNTRL n = 14 camundongos e WSMoL n = 11 camundongos), (B) peso corporal dos grupos CNTRL e WSMoL (CNTRL n = 14 camundongos e WSMoL n = 11 camundongos), (C) relação peso cardíaco/peso corporal (CNTRL n = 8 camundongos e WSMoL n = 7 camundongos) e (D) relação peso cardíaco/comprimento tibial, demonstrando que o tratamento com 5mg/kg de peso corporal de WSMoL preserva a estrutura cardíaca (CNTRL n = 8 camundongos e WSMoL n = 7 camundongos), (D) teste de tolerância à glicose intraperitoneal e (E) teste de tolerância à insulina intraperitoneal com seus gráficos de AUC correspondentes inseridos (CNTRL n = 9 camundongos e WSMoL n = 7 camundongos). Cada ponto representa valores individuais e as linhas representam valores médios. ○: Camundongos CNTRL; ●: Camundongos WSMoL. Foram realizadas as comparações entre grupos utilizando teste t de Student não pareado. Os resultados são mostrados como média ± desvio padrão.

O tratamento de 21 dias com WSMoL não induziu alterações (p > 0,05) nos níveis de glicose no sangue ( Figura 1A ) nem no peso corporal ( Figura 1B ), em comparação com o grupo CNTRL. Além disso as relações PC/PCorp ( Figura 1C ) e PC/CT ( Figura 1D ) foram semelhantes (p > 0,05) em ambos os grupos, indicando que nenhuma hipertrofia cardíaca foi desenvolvida. O tratamento também não modificou a tolerância à glicose ( Figura 1E ) ou a resistência à insulina ( Figura 1F ), em comparação com os camundongos não tratados (p > 0,05), revelando a ausência de alterações no metabolismo de carboidratos.

**Figura 2:**
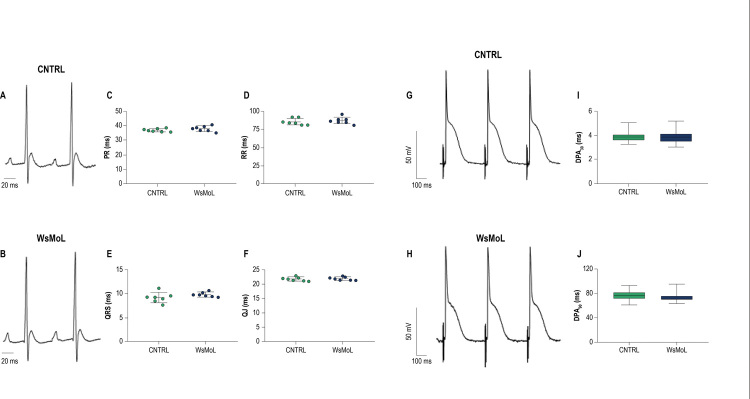
– WSMoL não comprometeu atividade elétrica cardíaca in vivo ou in vitro. Registros de ECG representativos in vivo dos grupos (A) CNTRL e (B) WSMoL. Os intervalos (C) PR, (D) RR, (E) QRS e (F) QJ sumarizaram os dados obtidos após 21 dias de tratamento com WSMoL (CNTRL n = 7 camundongos; 2.034 medições e WSMoL n = 7 camundongos; 2.038 medições). Cada ponto representa valores individuais e as linhas representam valores médios. Registros representativos in vitro do potencial de ação dos grupos (G) CNTRL e (H) WSMoL são mostrados. Os efeitos do tratamento com WSMoL na duração do potencial de ação (DPA) a (I) 30% e (J) 90% de repolarização são sumarizados (CNTRL n = 5 corações; 483 medições e WSMoL n = 4 corações; 545 medições). Cada ponto representa valores individuais e as linhas representam valores médios. Foram realizadas as comparações entre grupos utilizando teste t de Student não pareado, e dados que não apresentaram distribuição gaussiana (teste Kolmogorov-Smirnov) foram comparados pelo teste Mann-Whitney. ○: Camundongos CNTRL; ●: Camundongos WSMoL. Os resultados são mostrados como média ± desvio padrão para dados com distribuição gaussiana e como mediana e intervalo interquartil para dados com distribuição não gaussiana.

A Figura 2 mostra os parâmetros de ECG no 21º dia de tratamento. Os intervalos PR, RR, QRS e QJ ( Figura 2C–F ) não foram significativamente diferentes (p > 0,05) entre os grupos WSMoL e CNTRL. A DPA_30_ e a DPA_90_ foram semelhantes (p > 0,05) entre os camundongos tratados e não tratados ( Figura 2G–J ). Portanto, os dados obtidos aqui consistentemente demonstraram que o tratamento com WSMoL foi seguro para o comportamento elétrico de corações de camundongo.

**Figura 3:**
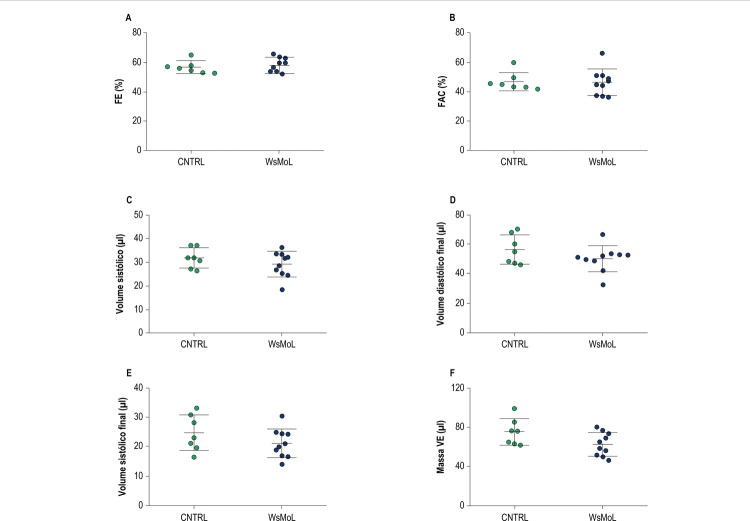
– Função e estrutura do ventrículo esquerdo estavam preservadas após tratamento com WSMoL. Os resultados obtidos por ECHO dos dois grupos estão sumarizados nos seguintes: (A) fração de ejeção ventricular, (B) alteração da área fracionária, (C) volume sistólico, (D) volume diastólico final (E) volume sistólico final e (F) massa ventricular esquerda (CNTRL n = 7 camundongos e WSMoL n = 10 camundongos). Foram realizadas as comparações entre grupos utilizando teste t de Student não pareado. Cada ponto representa valores individuais e as linhas representam valores médios. ○: Camundongos CNTRL; ●: Camundongos WSMoL. Os resultados são mostrados como média ± desvio padrão.

Considerando que tem sido demonstrado que alguns antibióticos podem prejudicar a função e a estrutura do ventrículo esquerdo, nós estudamos a função do ventrículo esquerdo detalhadamente utilizando ECHO ( Figura 3 ). Os camundongos dos grupos CNTRL e WSMoL apresentaram estrutura e função ventricular esquerda preservada, conforme indicada pela ausência de diferenças significativas (p > 0,05) nos parâmetros seguintes: fração de ejeção ( Figura 3A ), alteração da área fracionária ( Figura 3B ), volume sistólico ( Figura 3C ), volume diastólico final ( Figura 3D ), volume sistólico final ( Figura 3E ) e massa ventricular esquerda ( Figura 3F ). Como um conjunto, esses dados demonstram que o tratamento com WSMoL não prejudicou a função ventricular esquerda.

**Figura 4:**
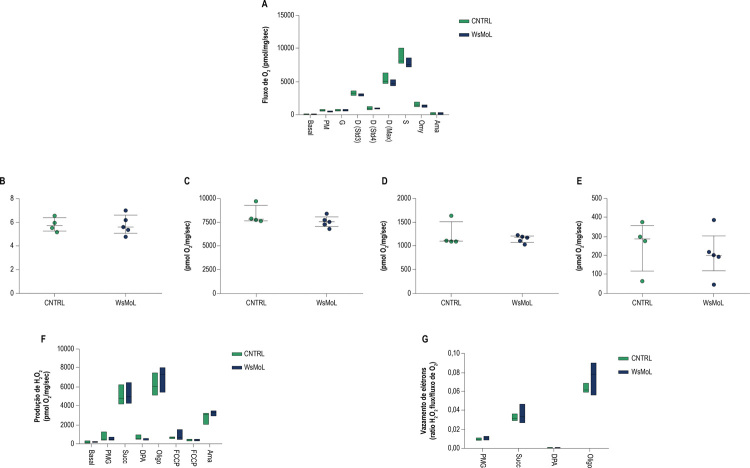
– WSMoL não alterou a função mitocondrial após 21 dias de tratamento. (A) Fluxos de consumo de O2 na respirometria de alta resolução dos grupos CNTRL e WSMoL, (B) relação de controle respiratório (RCR), (C) capacidade fosforilativa máxima do sistema de transporte de elétrons (OXPHOS), (D) vazamento inespecífico de prótons (LEAK), (E) consumo de oxigênio residual (ROX), (F) taxas de produção mitocondrial de H2O2 e (G) vazamento de elétrons nos grupos CNTRL e WSMoL. (CNTRL n = 4 corações e WSMoL n = 5 corações). Cada ponto representa valores individuais e as linhas representam valores médios. ○: Camundongos CNTRL; ●: Camundongos WSMoL. Foram realizadas as comparações entre grupos utilizando teste t de Student não pareado, e dados que não apresentaram distribuição gaussiana (teste Kolmogorov-Smirnov) foram comparados pelo teste Mann-Whitney. Os resultados são mostrados como média ± desvio padrão para dados com distribuição gaussiana e como mediana e intervalo interquartil para dados com distribuição não gaussiana.

Finalmente, para verificar se WSMoL interfere na fisiologia da função mitocondrial cardíaca, nós empregamos abordagens experimentais para analisar duas funções mitocondriais importantes: fosforilação oxidativa e produção de espécies reativas de oxigênio. O tratamento de 21 dias com WSMoL não induziu alterações no consumo de oxigênio mitocondrial, conforme demonstrado na Figura 4A–E . Além disso, o tratamento não interferiu com a taxa de produção de H_2_O_2_ na presença de diversos substratos, inibidores e desacopladores ( Figura 4F ), e não alterou o vazamento de elétrons ( Figura 4G ) em comparação com o grupo CNTRL.

## Discussão

A alta toxicidade de algumas drogas atualmente utilizadas para o tratamento várias doenças é uma grande preocupação em sistemas de saúde. Por exemplo, diversas classes de antibióticos são cardiotóxicas.^18 - 20^ Neste cenário, compostos naturais têm sido cada vez mais estudados devido ao seu potencial na descoberta e no desenvolvimento de medicamentos.^36^ Porém é importante também avaliar a segurança de compostos naturais utilizados para fins alimentares e médicos. Estudos anteriores realizados pelo nosso grupo demonstraram as atividades antibacterianas e imunomoduladoras da WSMoL,^8 - 10^ que também é uma proteína coagulante das sementes de *M. oleifera* . Neste estudo, nós avaliamos os potenciais efeitos cardiotóxicos da WSMoL, quando administrada por via oral em camundongos. Estudos de segurança são imperativos, mesmo quando as lectinas são administradas por via oral, visto que tem sido relatado que algumas proteínas desta classe podem atravessar a barreira intestinal e ser encontradas sistemicamente.^37^

Existe uma crença que a origem natural de um produto garanta a sua segurança em humanos. Porém alguns compostos naturais podem desencadear alguns efeitos tóxicos, inclusive no nível cardíaco. Por exemplo, o alcalóide aconitina, um ingrediente de Fuzi (um medicamento tradicional chinês), foi indicado como a causa de taquicardia ventricular Bidirecional.^38^

É também sabido que diversos antibióticos são capazes de bloquear os canais de potássio hERG, prolongando o intervalo QT e a DPA.^39 - 41^ Guo et al.,^42^ observaram o prolongamento da DPA utilizando eritromicina em miócitos ventriculares de camundongos recém-nascidos. Zhang et al.,^43^ também demonstraram que a azitromicina, quando administrada em porquinhos-da-índia, causou prolongamentos significativos das DPA_50_ e DPA_90_.

Neste sentido, nós avaliamos os efeitos do tratamento com WSMoL na atividade elétrica cardíaca tanto *in vivo* quanto *ex vivo* , em camundongos, observando a sua segurança cardiológica.

Outro efeito observado em alguns antibióticos é o comprometimento da função e estrutura ventricular esquerda, como observado por Zhang et al.,^43^ . Além disso, alguns estudos têm demonstrado que antibióticos e outros compostos naturais podem prejudicar a função mitocondrial.^44 , 45^ No entanto, após 21 dias de tratamento com WSMoL, nós observamos que a função ventricular esquerda e a função mitocondrial estavam preservadas.

## Conclusão

Os dados apresentados aqui indicam que a administração de WSMoL por gavagem não teve efeitos cardiotóxicos em camundongos tratados durante 21 dias. Estes resultados contribuem para a avaliação de segurança do uso de sementes para o tratamento da água, visto que essa lectina está presente na preparação empregada por algumas populações com este fim.
