# Supplementary material for: Avaliação dos Efeitos Cardíacos de Lectina Solúvel em Água (WSMoL) de Sementes de Moringa Oleifera
Source: Arq Bras Cardiol. 2020 Jun 29;114(6):1029–37. [Article in Portuguese] doi: 10.36660/abc.20190071 (PMC8416120; doi:10.36660/abc.20190071)
Supplement: Translation

# Translation: Evaluation of the Cardiac Effects of a Water-Soluble Lectin (Wsmol) from *Moringa Oleifera* Seeds

**Authors:** Ainhoa Rodríguez de Yurre, José Dayvid Ferreira da Silva, Marília Kalinne da Silva Torres, Eduarda Lopes Martins, Isalira Peroba Ramos, Wênio Sandoval Filho Lima da Silva, Jéssica da Silva Sarpa, Caio César da Silva Guedes, Thiago Henrique Napoleão, Luana Cassandra Breitenbach Barroso Coelho, Patrícia Maria Guedes Paiva, Emiliano Medei

**Affiliations:** 1 Brazil Universidade Federal do Rio de Janeiro, Rio de Janeiro, RJ – Brazil; 2 Brazil Universidade Federal de Pernambuco, Recife, PE – Brazil

**Keywords:** *Moringa Oleífera* (WSMoL), Lectins, Glycosides, Carbohydrates, Heart, Water Security, Mice

## Abstract

**Background:**

Moringa oleifera seeds, which are used for water clarification, contain a lectin named WSMoL which has shown in vitro antibacterial and immunomodulatory activity. Due to their nutritional value and therapeutic potential, the leaves and seeds of this tree are eaten in some communities. Some plant lectins are non-toxic to mammals, but others have been reported to be harmful when ingested or administered by other means.

**Objective:**

As one of the steps needed to define the safety of WSMoL, we evaluated possible cardiotoxic effects of this purified protein. Methods: WSMoL was administered for 21 consecutive days to mice by gavage. Electrophysiological, mechanical, and metabolic cardiac functions were investigated by in vivo and ex vivo electrocardiographic recordings, nuclear magnetic resonance, and high-resolution respirometry.

**Results:**

The treatment with WSMoL did not induce changes in blood glucose levels or body weight in comparison with control group. Moreover, the heart weight/body weight and heart weight/tibia length ratios were similar in both groups. Lectin ingestion also did not modify glucose tolerance or insulin resistance. No alterations were observed in electrocardiographic parameters or cardiac action potential duration. The heart of mice from the control and WSMoL groups showed preserved left ventricular function. Furthermore, WSMoL did not induce changes in mitochondrial function (in all cases, p > 0.05).

**Conclusions:**

The administration of WSMoL demonstrated a cardiac safety profile. These results contribute to the safety evaluation of using M. oleifera seeds to treat water, since this lectin is present in the preparation employed by some populations to this end. (Arq Bras Cardiol. 2020; [online].ahead print, PP.0-0)

## Introduction

*Moringa oleifera* Lamarck (Moringaceae) is a tree that is native to the south Himalaya region, widely cultivated on Asia and throughout the tropics mainly due to its use for water clarification. It has been employed in traditional medicine, as well as in food, cosmetic, and pharmaceutical industries,^1 , 2^ and it is also used to treat different diseases, such as cancer and chronic and infectious diseases.^3 , 4^

A water-soluble lectin isolated from the seeds of *M. oleifera* (WSMoL) has shown insecticidal activity,^5 - 7^ and *in vitro* studies demonstrated its antibacterial activity against corrosive and pathogenic bacteria.^8 - 10^ WSMoL demonstrated *in vitro* anti-inflammatory activity on lipopolysaccharide-stimulated murine macrophages,^11^ and it was able to activate human lymphocytes from peripheral blood mononuclear cell cultures, showing an immunomodulatory effect.^12^ It has also been proven that WSMoL is one of the coagulant proteins found in *M. oleifera* seeds^8 , 13^ and it is able to reduce the turbidity and ecotoxicity of water samples collected from a polluted stream.^14^

It is well demonstrated that many antibiotics and some classes of anti-inflammatory drugs are usually associated with cardiotoxic effects.^15 , 16^ The injurious events of these drugs in the cardiovascular system include the occurrence of heart failure with systolic ventricular dysfunction, arrhythmias, and myocardial ischemia.^17^ Classically, as a consequence of cardiotoxicity, changes on the electrocardiogram (ECG) can be observed, such as QT interval prolongation, which has been observed in patients who have used several classes of antimicrobials, including macrolides and fluoroquinolones.^18 - 20^ Among macrolides, intravenous administration of erythromycin presents the greatest risk. It increases the QT interval, and fatal arrhythmias have been reported when it was used alone or in combination with other QT-prolonging drugs.^16^ Thus, protection of cardiac function is currently a constant challenge for the pharmaceutical industry, regulatory authorities, and physicians facing adverse clinical reactions to various therapeutic agents in clinical practice.

WSMoL has emerged as a potential antibacterial drug and as an immunomodulatory agent. Some plant lectins are non-toxic to mammals,^21 , 22^ but others have been reported to be harmful when ingested or administered by other means, such as intraperitoneal injection.^23^ Thus, as one of the steps needed to define the safety of WSMoL, this study evaluated this protein’s possible cardiotoxic effects.

## Methods

### Plant material and lectin isolation

*Moringa oleifera* seeds were collected in Recife (Pernambuco, Brazil) with the authorization (no. 38690) of the Chico Mendes Institute for Biodiversity Conservation (ICMBio, acronym in Portuguese) and stored at −20 ºC. A sample of the collected material was stored as a voucher specimen (number 73345) at the Dárdano de Andrade Lima herbarium of the Agronomy Institute of Pernambuco. The access was recorded (A6CAB4C) in the National System For The Management Of Genetic Heritage And Associated Traditional Knowledge (SisGen, acronym in Portuguese).

WSMoL was isolated from seed powder according to the protocol previously described by Coelho et al.^5^ Briefly, proteins were extracted in distilled water, and, after filtration and centrifugation, the extract was treated with ammonium sulfate at 60% saturation^24^ for 4 h at 28 °C. After another centrifugation, the precipitate was resuspended in water and dialyzed for 8 h against distilled water (4 h) and 0.15 M NaCl (4 h). The dialyzed fraction (100 mg of proteins) was loaded onto a chitin column equilibrated with 0.15 M NaCl (20 mL/h flow rate) and WSMoL was eluted with 1.0 M acetic acid. The isolated lectin was dialyzed against distilled water with three liquid changes for eluent elimination. Carbohydrate-binding activity of the lectin was monitored during the purification process by the hemagglutinating activity assay according to the method described by Paiva and Coelho.^25^

### Animals

Adult male C57BL/6 mice were used and maintained at the Carlos Chagas Filho Biophysics Institute (IBCCF, acronym in Portuguese) of the Federal University of Rio de Janeiro (UFRJ, acronym in Portuguese) under controlled conditions of constant temperature (23 °C), in a standard light/dark cycle of 12h/12h with free access to food and water. All experiments were performed in accordance with the Ethical Principles in Animal Research adopted by the Brazilian College of Animal Experimentation, and the the applied protocols received approval from the Committee on Ethics in Animal Research of UFRJ, under protocol number DFBCICB041. The mice were used for experiments for 21 days.

### Experimental conditions

The animals were separated into two experimental groups: CNTRL (control group) and WSMoL (animals treated with WSMoL). Several studies by our group have extensively performed experiments with WSMoL using concentrations ranging from 10 μg/ml to 0.2 mg/ml^5 - 12^ in order to test several biological effects of WSMoL. In the present study, in order to test its cardiotoxicity of this purified protein, a 10 times higher concentration of WSMoL was used. Thus, the animals of the WSMoL group were treated with the lectin (purified protein) via gavage, at a concentration of 5 mg/kg body weight (equivalent to 2 mg/ml) for 21 days. The animals in the CNTRL group were treated with milli-Q water via gavage for 21 days.

### Cardiac hypertrophy

In order to evaluate the existence of possible cardiac hypertrophy, the mice’s hearts were weighed, and data were normalized by calculating the heart weight/body weight (HW/BW) and heart weight/tibia length (HW/TL) ratios.^26 , 27^ After weighing, the animals were euthanized by cervical dislocation. Subsequently, the hearts were extracted, washed with phosphate buffered saline (PBS), dried to remove liquid excess, and weighed. The length of the tibia was measured with a caliper.

### Fasting glucose, intraperitoneal glucose tolerance test, and intraperitoneal insulin tolerance test

Fasting blood glucose (FBG) concentrations were determined from tail vein blood using an automated glucometer (Contour^TM^TS Bayer). For intraperitoneal glucose tolerance test (IPGTT) and intraperitoneal insulin tolerance test (IPITT), mice were fasted for 6 h and 4 h, respectively. After the fasting period, animals received intraperitoneally 2 g/kg of glucose for IPGTT or 0.5 IU/kg of insulin for IPITT,^28^ and FBG levels were monitored 0, 15, 30, 60, 120 min after injection from a tail snip. The area under the curve (AUC) was calculated using all the time points, discounting baseline glucose values from each animal.

### Electrocardiography and echocardiography

In order to assess the cardiac electrical activity *in vivo* , an electrocardiogram (ECG) recording was carried out in conscious animals using a noninvasive method,^29^ namely, two subcutaneous electrodes were implanted under isoflurane anesthesia in the right and left forepaws, corresponding to ECG lead I. At the time of registration, the electrodes were connected by flexible cables to a homemade DC-coupled differential amplifier (kindly provided by Dr. Ariel Escobar, University of California, Merced, USA) using a 500 Hz low-pass filter and an acquisition frequency of 1 kHz. The signal was digitized using Digidata 1440A (Axon Instruments, San José, CA, USA) and recorded using a Labview-based acquisition program (National Instruments, Austin, TX, USA). The durations of the following intervals were analyzed: PR, RR, QRS, and QJ.

Cardiac function was evaluated by *in vivo* echocardiography (ECHO) using the Vevo 770 High-Resolution Imaging System (VisualSonics, Toronto, Canada) coupled to a 30 MHz transductor, under isoflurane anesthesia. Images were acquired in bidimensional mode and analyzed by a blinded investigator. Left ventricular end-diastolic volume, end-systolic volume, ejection fraction, and fractional area change were calculated using Simpson’s method. In brief, these parameters of cardiac function were evaluated in a long parasternal axis view and four high-temporal resolution B-mode short-axis images, taken at different ventricular levels, as described previously.^30^

### Action potential

In order to perform intact cardiac action potential (AP) records, a Langendorff retrograde perfusion system was used to maintain the hearts functional *ex vivo* for hours, as previously described.^31 , 32^ To avoid tissue damage by the formation of blood clots, animals were injected intraperitoneally with Na^+^-heparin 15 min before euthanization by cervical dislocation. Hearts were rapidly removed, cannulated by the aorta, and perfused continuously with an oxygenated Tyrode solution containing the following, (in mM): NaCl 140, KCl 5.3, CaCl_2_2, MgCl_2_1, NaPO4H2 0.33, HEPES 10, and glucose 10. The pH was calibrated to 7.4 with NaOH at 32 ºC. To decrease mechanical contraction, the hearts were perfused with Tyrode containing 4 mM of Blebbistatin (Selleckchem, Houston, TX, USA).

To record electrical signals, borosilicate glass (10-40 MΩ) microelectrodes were used. These microelectrodes were filled with 3 M KCl solution and inserted into a holder (MEH1SF12, World Precision Instrument [WPI], Sarasota, FL, USA) embedded in a micromanipulator (MM33 links, WPI) connected to the input of a pre-amplifier (Electro 705, WPI). The microelectrodes were placed on the surface of the left ventricle and the reading of the microelectrode was set to zero. Amplified signals were digitalized (NI USB 6281, National Instrument) and analyzed with a homemade program in LabView (kindly developed and provided by Dr. Ariel Escobar, University of California, Merced, CA, USA).

The parameters analyzed were action potential duration (APD) at 30% and 90% repolarization (APD_30_and APD_90_, respectively).

### Isolation of mice heart mitochondria

Isolation of mitochondria from the hearts was adapted from the protocol described by Affourtit et al.^33^ with minor modifications. The hearts were rapidly dissected and rinsed in ice-cold Chappell-Perry (CP) buffer containing the following (in mM): KCl 100, Tris-HCl 50, EGTA 2 at pH 7.2). The hearts were weighed, minced with razor blades and washed 4 to 5 times with CP buffer. The tissue was subsequently incubated for 5 min with CP buffer supplemented with 0.5% albumin, 5 mM MgCl2, 1 mM ATP, and 125 U/100 mL protease type VIII, at a proportion of 1 mL/100 mg of tissue. The hearts were then homogenized (Ultra-turrax homogenizer [IKA®, Campinas, SP, Brazil], low setting, 3 s, 3 times) and the resultant homogenate was centrifuged. The supernatant was centrifuged and the pellet was washed and resuspended into ice-cold CP buffer and finally centrifuged. The final mitochondrial pellet was resuspended into a small volume of CP buffer. The protein dosage of the obtained preparation was performed by the method described by Lowry et al.^34^ . The isolated mitochondrial preparations were subjected to high resolution respirometry to measure the fluxes of oxygen consumption.

### High resolution respirometry

For the analyses of the oxygen consumption, isolated mitochondria were used. The experiments were performed on a high-resolution O2k-respirometer (Oroboros Instruments, Innsbruck, Austria, EU) at 37°C with mitochondrial respiration media (MIR05) containing the following (in mM): EGTA 0.5, MgCl_2_3, K-MES 60, taurine 20, KH_2_PO_4_20, HEPES 20, sucrose 110 and 1 g/L fat free BSA at pH 7.1. The protocol used to evaluate mitochondrial function was adapted from Pesta and Gnaiger,^35^ consisting of sequential addition of multiple substrates and inhibitors, namely, the following: 5 mM pyruvate, 2.5 mM malate, 10 mM glutamate, 100 μM adenosine 5’-diphosphate (ADP), 1 mM ADP, 10 mM succinate, 0.2 μg/mL oligomycin, and 2 μM antimycin A. Respiratory control ratio (RCR) was calculated by the oxygen flux after addition of succinate in the presence of ADP, divided by the flux after oligomycin. The maximal phosphorylative capacity of electron transport system (OXPHOS) was calculated by the oxygen consumption following addition of succinate minus residual oxygen consumption (ROX), which was estimated after the addition of antimycin A. The non-specific leak of protons was determined by oxygen flux insensitive to oligomycin minus ROX. Another distinct protocol was performed by changing the sequence of the substrates in order to calculate the electron leakage, the ratio of hydrogen peroxide (H_2_O_2_) production by O_2_flux. The order of titration of this protocol was the following: 5 mM pyruvate, 2.5 mM malate, 10 mM glutamate, 10 mM succinate, 1 mM ADP, and 0.2 μg/mL oligomycin. Data were analyzed in DatLab 5 software (Oroboros Instruments) and expressed in pmol O_2_/mg/s.

### Mitochondrial H2O2 production

Mitochondrial H_2_O_2_was measured by monitoring the resorufin appearance rate at 563/587 nm (excitation/emission) in a fluorescence spectrophotometer (Varian Cary Eclipse, Agilent Technologies, Santa Clara, CA, USA). The same concentration of isolated mitochondria used in the oxygen consumption experiments was added in 2 mL of MIR05 supplemented with 5.5 μM Amplex red, 2 U/mL peroxidase, and 40 U/mL superoxide dismutase. The assays of H_2_O_2_production were performed at 37 ºC, and the substrates, inhibitors, and uncouplers were added every 2 min in the following order: 5 mM pyruvate, 2.5 mM malate, 10 mM glutamate, 10 mM succinate, 1 mM ADP, 0.2 μg/mL oligomycin, 2 titres of 0.5 μM carbonyl cyanide-4-(trifluoromethoxy) phenylhydrazone (FCCP), and 2 μM antimycin A. The data generated in arbitrary units of fluorescence were analyzed in Origin Pro-8 software (Origin Lab Corporation, Northampton, MA, USA) and normalized to pmol of H_2_O_2_/mg/min from standard calibration curves of H_2_O_2_performed in the presence of the same number of isolated mitochondria for each experiment.

### Statistical analysis

Values are expressed as mean ± SD or median (with interquartile range). In order to compare the results between CNTRL and WSMoL, unpaired Student’s t test was used, when appropriate. On the other hand, data showing non-Gaussian distribution (Kolmogorov-Smirnov test) were compared by the Mann-Whitney test. Differences between variables were considered significant when p value was < 0.05. All analyses were performed using GraphPad Prism 7.0 (GraphPad Software, San Diego, CA, USA). We did not use statistical methods to predetermine sample size. Samples sizes were estimated on the basis of sample availability and previous experimental studies of the cardiovascular system.^29 , 30^

## Results

The 21-day treatment with WSMoL did not induce changes (p > 0.05) in blood glucose levels ( Figure 1A ) or body weight ( Figure 1B ) in comparison with CNTRL group. In addition, the HW/BW ( Figure 1C ) and HW/TL ( Figure 1D ) ratios were similar (p > 0.05) in both groups, indicating that no cardiac hypertrophy was developed. The treatment also did not modify glucose tolerance ( Figure 1E ) or insulin resistance ( Figure 1F ) in comparison with untreated mice (p > 0.05), revealing absence of alterations in carbohydrate metabolism.

**Figure 1:** – WSMoL treatment for 21 days did not induce metabolic alterations. (A) Blood glucose levels after 21 days of treatment with saline (CNTRL) or WSMoL (WSMoL) solution (CNTRL n = 14 mice and WSMoL n = 11 mice), (B) body weight of CNTRL and WSMoL groups (CNTRL n = 14 mice and WSMoL n = 11 mice), (C) heart weight/body weight ratio (CNTRL n = 8 mice and WSMoL n = 7 mice) and (D) heart weight/tibia length ratio, showing that the treatment with 5mg/kg body weight of WSMoL preserves cardiac structure (CNTRL n = 8 mice and WSMoL n = 7 mice), (D) intraperitoneal glucose tolerance test and (E) intraperitoneal insulin tolerance test with their correspondent AUC graphs on inset (CNTRL n = 9 mice and WSMoL n = 7 mice). Each dot represents individual values and lines represent mean values. ○: CNTRL mice; ●: WSMoL mice. Comparisons between groups were performed using unpaired Student’s t test. The results are shown as mean ± SD.

Figure 2 shows the ECG parameters at day 21 of treatment. The PR, RR, QRS, and QJ intervals ( Figure 2C–F ) were not significantly different (p > 0.05) between the WSMoL and CNTRL groups. The APD_30_and APD_90_were similar (p > 0.05) between untreated and treated mice ( Figure 2G–J ). Thus, the data obtained here consistently demonstrated that WSMoL treatment was safe for the electrical behavior of mouse heart.

**Figure 2:** – WSMoL did not impair in vivo or in vitro cardiac electrical activity. Representative in vivo ECG recordings of (A) CNTRL and (B) WSMoL groups. (C) PR, (D) RR, (E) QRS and (F) QJ intervals summarized the data obtained after 21 days of WSMoL treatment (CNTRL n = 7 mice; 2,034 measurements and WSMoL n = 7 mice; 2,038 measurements). Each dot represents individual values and lines represent mean values. Representative in vitro recordings of ventricular action potential of (G) CNTRL and (H) WSMoL groups are showed. The effect of WSMoL treatment on action potential duration at (I) 30% and (J) 90% repolarization are summarized (CNTRL n = 5 hearts; 483 measurements, and WSMoL n = 4 hearts; 545 measurements). Each dot represents individual measurements and lines represent mean values. Comparisons between groups were performed using unpaired Student’s t test, and data that did not show Gaussian distribution (Kolmogorov-Smirnov test) were compared by the Mann Whitney test. ○: CNTRL mice; ●: WSMoL mice. The results are shown as mean ± SD for data with Gaussian distribution and as median and interquartile range for data with non-Gaussian distribution.

Since some antibiotics have been shown to be able to impair left ventricular function and structure, we studied the left ventricular function in detail by ECHO ( Figure 3 ). The mice from both CNTRL and WSMoL groups showed preserved left ventricular structure and function, as indicated by the absence of significant differences (p > 0.05) in the following parameters: ejection fraction ( Figure 3A ), fractional area change ( Figure 3B ), stroke volume ( Figure 3C ), end-diastolic volume ( Figure 3D ), end-systolic volume ( Figure 3E ), and left ventricular mass ( Figure 3F ). Taken together, these data show that WSMoL treatment did not impair left ventricular function.

**Figure 3:** – Left ventricular function and structure were preserved after WSMoL treatment. The results obtained by ECHO from both groups are summarized in the following: (A) ventricular ejection fraction, (B) fractional area change, (C) stroke volume, (D) final diastolic and (E) final systolic volume, and (F) left ventricular mass (CNTRL n = 7 mice and WSMoL n = 10 mice). Comparisons between groups were performed using unpaired Student’s t test. Each dot represents individual values and lines represent mean values. ○: CNTRL mice; ●: WSMoL mice. The results are shown as mean ± SD.

Finally, in order to verify whether WSMoL interferes in the physiology of heart mitochondrial function, we performed experimental approaches to analyze two important mitochondrial functions: oxidative phosphorylation and reactive oxygen species production. The 21-day treatment with WSMoL did not induce alterations in the mitochondrial oxygen consumption, as can be observed in Figure 4A–E . In addition, the treatment did not interfere in the rate of H_2_O_2_production in the presence of different substrates, inhibitors, and uncouplers ( Figure 4F ), nor did it alter electron leakage ( Figure 4G ) when compared to the CNTRL group.

**Figure 4:** – WSMoL did not alter mitochondrial function after 21 days of treatment. (A) O2 consumption fluxes in high-resolution respirometry of CNTRL and WSMoL groups, (B) respiratory control ratio (RCR), (C) maximal phosphorylative capacity of electron transport system (OXPHOS), (D) non-specific leak of protons (LEAK), (E) residual oxygen consumption (ROX), (F) rates of mitochondrial H2O2 production, and (G) electron leakage of CNTRL and WSMoL groups. (CNTRL n = 4 hearts and WSMoL n = 5 hearts). Each dot represents individual values and lines represent mean values. ○: CNTRL mice; ●: WSMoL mice. Comparisons between groups were performed using unpaired Student’s t test, and data that did not show Gaussian distribution (Kolmogorov-Smirnov test) were compared by the Mann Whitney test. The results are shown as mean ± SD for data with Gaussian distribution and as median and interquartile range for data with non-Gaussian distribution.

## Discussion

The high toxicity of some drugs currently used for treatment of several diseases is a major concern in health systems. For example, several classes of antibiotics are cardiotoxic.^18 - 20^ In this scenario, natural compounds have been increasingly studied due to their potential for drug discovery and development.^36^ However, it is also important to evaluate the safety of natural compounds used for food and medical purposes. Previous studies by our group demonstrated the antibacterial and immunomodulatory activities of WSMoL,^8 - 10^ which is also a coagulant protein from *M. oleifera* seeds. In this study, we evaluated possible cardiotoxic effects of orally administrated WSMoL on mice. Safety studies are imperative, even when lectins are administered orally, since it has been reported that some proteins of this class can cross the intestinal barrier and be found systemically.^37^

There is a belief that the natural origin of a product guarantees its safety to humans. However, some natural compounds may exert toxic effects, including on the cardiac level. For example, the alkaloid aconitine, an ingredient of Fuzi (a traditional Chinese medicine), was pointed out as the cause of bidirectional ventricular tachycardia.^38^

It is also well known that several antibiotics are able to block hERG potassium channels, prolonging the QT interval and the APD.^39 - 41^ Guo et al.^42^ observed a prolongation of APD using erythromycin in neonatal mouse ventricular myocytes. Zhang M. et al.^43^ also showed that azithromycin, when administered in guinea pigs, caused significant prolongations of APD_50_and APD_90_.

Accordingly, we evaluated the effects of WSMoL treatment on the cardiac electric activity both *in vivo* and *ex vivo* , in mice, observing that it was cardiologically safe.

Another effect observed in some antibiotics is the impairment of left ventricular function and structure, as observed by Zhang M. et al.^43^ . Furthermore, some studies have shown that antibiotics and other natural compounds can impair mitochondrial function.^44 , 45^ However, after 21 days of WSMoL treatment, we observed that left ventricular function and mitochondrial function were preserved.

## Conclusion

The data presented here indicate that the administration of WSMoL by gavage did not have cardiotoxic effects on mice treated for 21 days. These results contribute to the safety evaluation of the use of *M. oleifera* seeds to treat water, since this lectin is present in the preparation employed by some populations to this end.
